# Physical activity, sports participation, and sustainable development in the Ibero-American region: a pilot implementation of indicators in Chile, Costa Rica, and Ecuador

**DOI:** 10.3389/fspor.2025.1607167

**Published:** 2025-09-17

**Authors:** Robert Bauer, Raúl Sánchez-García, Daniela Hernández, Domingo F. Hernández-Angeles, Silvia González, Inés Nieto, Xián Mayo, Alfonso Jiménez

**Affiliations:** ^1^ Research Centre in Sports Science (CIDE), King Juan Carlos University, Madrid, Spain; ^2^ Ibero-American Sports Council, Madrid, Spain; ^3^ UNESCO, Montevideo, Uruguay; ^4^ District Institute of Recreation and Sports, Bogota, Colombia

**Keywords:** evaluation, framework, health, policy coordination, monitoring, United Nations 2030 Agenda, Ibero-American indicators model

## Abstract

**Background:**

In recognition of the potential contributions of sports and physical activity to national development and responding to international calls from organizations like UNESCO and the World Health Organization, a coalition of agencies and Ibero-American sports ministers endorsed the creation of an indicator set to assess the impact of sports participation and physical activity on sustainable development.

**Methods:**

The Ibero-American Sports Council, UNESCO, and the German Agency for International Cooperation developed a set of twelve Sports and Development indicators and four key data points for the Ibero-American region. In 2024, the first national-level pilot implementation was completed in Chile, Costa Rica, and Ecuador, collecting data using specifically designed formulae.

**Results:**

Each country was able to partially provide relevant data for the defined indicators, with Chile completing eight, Costa Rica four, and Ecuador seven indicators. All countries provided data for the key data points.

**Conclusions:**

The first national pilot implementation of the proposed indicators—as part of its validation process—highlights the need for improved quality and accessibility of reliable data across the Ibero-American region. These indicators have the potential to assess, track, and compare policies related to physical activity and sports, and to identify challenges and opportunities for improvement.

## 1 Introduction

Sports and physical activity play a critical role in fostering national development by empowering vulnerable populations and promoting health, education, and social inclusion (1–5). However, there is a need for more systematic efforts to monitor and evaluate the impact of policies that promote physical activity within public health strategies (6).

The World Health Organization (WHO) defines *physical activity* as “any bodily movement produced by skeletal muscles that requires energy expenditure” (7, 8). Its measurement can then be used to classify sufficient and insufficient levels. Thus, *physical inactivity* describes “an insufficient physical activity level to meet present physical activity recommendations” (8, 9). Finally, the term *sport* encompasses numerous “activities performed within a set of rules and undertaken as part of leisure or competition. Sporting activities involve physical activity carried out by teams or individuals and may be supported by an institutional framework, such as a sporting agency” (8).

The positive effects of regular physical activity and sports on health are significant, potentially reducing the risk of all-cause mortality, cardiovascular disease mortality, and the incidence of coronary heart disease, stroke, hypertension, type 2 diabetes, cancer, dementia, and depression. Additional positive effects may include increases in mental health, quality of life, and well-being (10–15).

In Latin America, studies showed that 39% of adults and 85% of adolescents fail to meet recommended physical activity levels (16–18). Furthermore, research indicates significant gender disparities in physical activity levels for all age groups. Among adults, findings from the Latin American Study of Nutrition and Health indicate that men participate in greater amounts of moderate-to-vigorous and overall physical activity compared to women. Thus, the prevalence of physical inactivity was notably higher among women (47.7%) compared to men (33.0%; 17). Likewise, a study from the South American Physical Activity and Sedentary Behavior Network (19), which analyzed physical activity and sedentary behavior across six South American countries, found that men were more active in leisure and occupational activities, whereas women engaged more in transportation-related physical activity. Despite this, women had higher overall rates of physical inactivity. Additionally, higher educational attainment was linked to lower levels of physical activity among men and younger adults, but this association was not observed for women or for middle-aged and older adults (19).

Among adolescents, research also reveals gender disparities. Salvo et al. (20) found that Brazilian boys are more physically active than girls, reflecting the influence of both biological and sociocultural factors. This aligns with findings from a large-scale study involving over 200,000 Latin American children and adolescents, which found that boys are more likely than girls to meet the WHO's recommended minimum of 60 min of moderate-to-vigorous physical activity daily (21). Overall, these findings emphasize the importance of targeted interventions to encourage physical activity, particularly among women, in the Ibero-American region.

Beyond these health-related benefits, physical activity and sport can advance national development by promoting education, inclusion, peace, and equity (22, 23). Within the framework of the 2030 Agenda for Sustainable Development, they are recognized as contributing to at least ten of the seventeen Sustainable Development Goals (SDGs; 5, 15, 24, 25). Specifically, participation in physical activity and sport contributes to improved health outcomes by reducing non-communicable diseases and enhancing mental health (SDG3; 26), and is associated with better educational outcomes (SDG4). Additionally, sport policies have the potential to reduce gender discrimination (SDG5), promote decent work and economic growth (SDG8), and foster social, economic, and political inclusion (SDG10). Also, active commuting strategies can support the development of sustainable transport systems (SDG11) and contribute to environmental sustainability and responsible consumption (SDG12 and 13). Furthermore, many sports and physical activity initiatives indirectly encourage sustainable land use and conservation (SDG15) and can help reduce violence and conflict (SDG16; 15, 24, 27–30).

Considering this potential impact of physical activity and sports on the development of nations, measuring initiatives have emerged in response to international calls to create common indicators such as the Kazan Action Plan (28). More specifically, the Commonwealth Secretariat has been actively exploring the connection between sport and sustainable development, measuring sport's contribution to the SDGs (31–33). They have developed a comparative content analysis of public sports policies in 40 countries worldwide, aiming to evaluate the degree of alignment between national sport policies and the SDGs. They have also produced a toolkit on sport and SDG indicators (34). The Commonwealth's indicators model, based on the conceptual framework of the theory of change, includes three levels of indicators: the first level pertains to a national scope; the second one, more specific, pertains to local and regional levels; and the third, the programmatic level, pertains to interventions based on projects (34).

For the Ibero-American region, Ministers and High Authorities of Sport advocated for the creation of a feasible system of indicators to measure the impact of physical activity and sports on sustainable development in the context of the region (35). For this purpose, a set of indicators was developed through a participatory process led by the Ibero-American Sports Council (CID for its acronym in Spanish), UNESCO, and the German Agency for International Cooperation (GIZ for its acronym in German). The indicators were designed to: a) encourage countries to collect periodic, standardized, and comparable information on the impact of sports on various dimensions of sustainable development, such as health, education, gender equity, inequalities, peace, and social cohesion; b) provide relevant information to estimate the social return on investment in sport; and c) support the periodic evaluation of sport-related policies to identify areas for improvement and recognize achievements (35, 36).

The Ibero-American set of twelve indicators and four key data points was developed based on the conceptual frameworks such as those of the Commonwealth (34, 37), the World Health Organization (15), and the Global Observatory for Physical Activity (GoPA!; 38). While grounded in these established models, the initial selection was adapted to the specific realities of the Ibero-American region, where sport- and physical activity-related data are often scarce or just emerging. The indicators were intended to support countries in monitoring the contribution of sport and physical activity to sustainable development through a simplified and policy driven approach.

The development process prioritized six thematic areas aligned with the SDGs—health (SDG 3), education (SDG 4), gender equality (SDG 5), economy (SDG 8), inclusion (SDG 10), and peace (SDG 16)—and involved experts and government representatives from across the region. Through multiple iterations, including focus groups and interviews, stakeholders assessed and refined the indicators based on relevance, feasibility, and regional priorities. The final set of indicators and key data points, along with their pilot implementation, was formally approved at the CID General Assembly in 2023. Chile, Costa Rica, and Ecuador—member countries of CID—were selected to conduct the first pilot implementation (36, 39). As summarized in Table 1, all selected indicators deliver meaningful measurements expressed as percentages. This format ensures clear interpretability and facilitates straightforward comparability across different datasets or contexts. Additionally, percentage-based indicators provide an intuitive way to communicate results to diverse audiences, support benchmarking efforts, and enhance decision-making through standardized metrics.

**Table 1:** The Ibero-American indicators for sport and sustainable development.

Development area	Indicator	Estimation formula
Economy (SDG 8)	1. Percentage of the national public budget invested in sports and physical activity	• $PBISPA=\left(\frac{BISPA}{NPB}\right)100$ • PBISPA = Percentage of the national public budget invested in sports and physical activity • BISPA = National public budget invested in sports and physical activity • NPB = National public budget. It is the monetary and financial amount for the operation of a government in a given period (normally one year), calculated, registered, and published by the countries' ministries of finance
	2. Percentage of the contribution of sports and physical activity to the national GDP	• $PSGDP=\left(\frac{SGDP}{GDP}\right)100$ • PSGDP = Percentage of the contribution of sports and physical activity to the national GDP • SGDP = Monetary value of all goods and services related to sports and physical activity produced in a country in a given period (normally one year) • GDP = Gross Domestic Product. It is equal to the monetary value of all goods and services produced in a country or territory during a period (normally one year)
Health (SDG 3)	3. Percentage of the population sufficiently physically active stratified by gender and age	• $PPSA=\left(\frac{PSA}{TP}\right)100$ • PPSA = Percentage of population sufficiently physically active • PSA = Population sufficiently physically active • TP = Total population
	4. Percentage of deaths related to physical inactivity	• $par_{sem1}=1-\frac{1}{(1-p_{1})+p_{1}rr_{1}^{\left(a\right)}}$ • par_sem1_ = semi-adjusted population attributable risk • $rr_{1}^{\left(a\right)}$ = 1.28^a^ corresponds to the multivariate-adjusted relative risk of all-cause mortality due to physical inactivity^b^ • p_1_ = prevalence of insufficient physical activity
Education (SDG 4)	5. Percentage of schools providing the minimum number of minutes of physical education	• $PSMPE=\left(\frac{SMPE}{TS}\right)100$ • PSMPE = Percentage of schools providing the minimum number of minutes of physical education • SMPE = Schools providing the minimum number of minutes of physical education (120/180 min per week depending on the grade) • TS = Total number of schools
	6. Percentage of school students sufficiently physically active stratified by gender	• $PSSA=\left(\frac{SSA}{TS}\right)100$ • PSSA = Percentage of school students sufficiently physically active • SSA = School students sufficiently physically active • TS = Total number of school students
Gender equality (SDG 5)	7. Percentage of the national sport entity's budget focused on gender programs	• $PBG=\left(\frac{BG}{BNS}\right)100$ • PBG = Percentage of the national sport entity's budget focused on gender programs • BG = Budget of the national sports entity focused on gender programs • BNS = Budget of the national sports entity
	8. Percentage of women in managerial positions within the national sports entity	• $PWMP=\left(\frac{MPW}{TMP}\right)100$ • PWMP = Percentage of women in managerial positions within the national sports entity • MPW = Managerial positions held by women within the national sports entity • TMP = Total number of managerial positions in the national sports entity
	9. Percentage of the gender gap among the sufficiently physically active population	• $PGGP=\left(\frac{\left(PM-PW\right)}{PM}\right)(100)$ • PGGP = Percentage of the gender gap among the sufficiently physically active population • PM = Percentage of physically active men • PW = Percentage of physically active women
Inclusion (SDG 10)	10. Percentage of the national sport entity's budget focused on programs for people with disabilities	• $PNBPD=\left(\frac{NBPD}{NB}\right)100$ • PNBPD = Percentage of the national sport entity's budget focused on programs for people with disabilities • NBPD = National sport entity's budget focused on programs for people with disabilities • NB = National sport entity's budget
	11. Percentage of the disability gap among the sufficiently physically active population	• $PDG=\left(\frac{\left(PPND-PPD\right)}{PPND}\right)(100)$ • PDG = Percentage of the disability gap among the sufficiently physically active population • PPND = Percentage of sufficiently physically active people with no disabilities • PPD = Percentage of sufficiently physically active people with disabilities
Peace and social cohesion (SDG 16)	12. Percentage of government programs using sport as an instrument for peace and coexistence	• $PPPC=\left(\frac{NPPC}{TNP}\right)100$ • PPPC = Percentage of government programs using sport as an instrument for peace and coexistence • NPPC = Number of government programs using sport as an instrument for peace and coexistence • TNP = Total number of government programs

^a^ Lee et al. (11).

^b^ Wong et al. (40).

In comparison, both the Commonwealth model and Ibero-American indicators model promote periodic, standardized, and, therefore, comparable data acquisition and analysis regarding the influence of physical activity and sports across the various aspects of sustainable development, which also permits assessment of sports-related policies to identify potential areas for improvement. Both models present distinct approaches aimed at addressing the specific needs of the context in which an indicator model must be implemented for the targeted group of countries. As such, the Ibero-American indicators model may provide a more tailored analysis that better aligns with the unique needs and contexts of the Ibero-American region.

Regarding the ongoing development of the Ibero-American Indicators Model of Physical Activity and Sport for Sustainable Development, the King Juan Carlos University (Spain) and the Development Bank of Latin America and the Caribbean also joined the Consortium. Following its formal adoption during the XXIX General Assembly of CID in May 2023 (39), the model was piloted in two phases: first in Chile, Costa Rica, and Ecuador (September 2023 to April 2024), and then in Colombia and Peru (October 2024 to March 2025; 36).

The objectives of this pilot study were: a) to collect relevant data on sports, physical activity, and sustainable development using a standardized approach; b) to assess the current national efforts in sports and physical activity and their role in advancing sustainable development; c) to identify opportunities to strengthen policies and strategies that support sustainable development; and d) to highlight data gaps and research needs that must be addressed to better understand the role of sport and physical activity in sustainable development.

In this article, we present and analyze the results of the first implementation of the Ibero-American indicators for sports and sustainable development in Chile, Costa Rica, and Ecuador, as well as compare and discuss the current strengths and limitations of the Ibero-American indicator set.

## 2 Methods

Over several months, the consulting team collaborated with the technical teams of the three countries to identify relevant sources of data for the indicators and key data points specific to each nation. The main sources of data included surveys, national budgets, and policy documents (see Table 2).

**Table 2:** Data sources used for indicators by country.

Indicator	Chile	Costa Rica	Ecuador
1. Percentage of the national public budget invested in sports and physical activity	Law 21.516. Public Sector Budget 2023 (41)	Not reported	Public National Budget 2023 (42)
2. Percentage of the contribution of sports and physical activity to the national GDP	Not reported	Not reported	Not reported
3. Percentage of the population sufficiently physically active stratified by gender and age	2018 National Survey of Physical Activity and Sports Habits in the Population Aged 18 and over (43)	Surveillance of Cardiovascular Risk Factors, Third Survey 2018 (44)	Physical Activity Component of the Employment, Unemployment and Underemployment Survey ENEMDU 2022 (45)
4. Percentage of deaths related to physical inactivity	Chile Country Card of the Global Observatory of Physical Activity (38)	Costa Rica Country Card of the Global Observatory of Physical Activity (38)	Ecuador Country Card of the Global Observatory of Physical Activity (38)
5. Percentage of schools providing the minimum number of minutes of physical education	Not reported	Not reported	Not reported
6. Percentage of school students sufficiently physically active stratified by gender	2019 National Survey of Physical Activity and Sport in Children Aged 5–17 (46)	Not reported	Not reported
7. Percentage of the national sport entity's budget focused on gender programs	Law 21.516. Public Sector Budget 2023 (41)	Not reported	Not reported
8. Percentage of women in managerial positions within the national sports entity	Report on Gender Distribution In Management Positions in the Sports Sector (47)	Report of Executive Heads of Unit and/or Department at the Costa Rican Institute of Sport and Recreation (48)	Staff distribution of the Ministry of Sports of Ecuador for 2023 (49)
9. Percentage of the gender gap among the sufficiently physically active population	2018 National Survey of Physical Activity and Sports Habits in the Population Aged 18 and over (43)	Surveillance of Cardiovascular Risk Factors, Third Survey 2018 (44)	Physical Activity Component of the Employment, Unemployment and Underemployment Survey ENEMDU 2022 (45)
10. Percentage of the national sport entity's budget focused on programs for people with disabilities	Not reported	Not reported	Budget distribution for sports programs targeted to people with disabilities 2023 (50)
11. Percentage of the disability gap among the sufficiently physically active population	Not reported	Not reported	Not reported
12. Percentage of government programs using sport as an instrument for peace and coexistence	Integrated bank of social and non-social programs (BIPS) 2024 (51)	Not reported	National Project Sports for Development 2022–2025 (52)

We conducted a secondary analysis of this data using predefined methodologies specified in technical data sheets (53). These data sheets outlined their key characteristics and attributes, ensuring clear interpretation, effective implementation, and scalability. As such, they play a crucial role in replicating indicator calculations, defining their scope, fostering expert discussions, and enhancing information transparency. As a summary of this information, Table 1 presents the formulae used to calculate indicator values for each country, where applicable (for more information, see 53). For Indicator I1, countries reported the percentage of the public budget allocated to physical activity and sport, based on their respective national budget structures. I3, I6, and I9 were informed by each country's most recent national surveys on physical activity or cardiovascular risk factor surveillance. I4 was based on the country profiles published in the *Second Physical Activity Almanac* by GoPA! (38). For I7 and I10, data were obtained from specific policy documents that included budget breakdowns. I12 was informed by national physical activity and sport policies. No data sources were reported for I2, I5, and I11.

## 3 Results

All three countries provided information for each of the four key data points (that is, 100% implementation). However, when assessed against the criteria outlined in the technical data sheets (53), each country's findings exhibited limitations (54–56), resulting in a *partially* rating in several cases (see Table 3).

**Table 3:** Indicator and Key data point results for each country.

Indicator	Chile	Costa Rica	Ecuador
1. Percentage of the national public budget invested in sports and physical activity	0.62%	–	0.23%
2. Percentage of the contribution of sports and physical activity to the national GDP	–	–	–
3. Percentage of the population sufficiently physically active stratified by gender and age	18.7%	63.9%	78.3%
4. Percentage of deaths related to physical inactivity	8.3%	11.4%	11.0%
5. Percentage of schools providing the minimum number of minutes of physical education	–	–	–
6. Percentage of school students sufficiently physically active stratified by gender	16.5%	–	–
7. Percentage of the national sport entity's budget focused on gender programs	0%	–	–
8. Percentage of women in managerial positions within the national sports entity	52.4%	36.4%	48.6%
9. Percentage of the gender gap among the sufficiently physically active population	42.9%	15.3%	17.0%
10. Percentage of the national sport entity's budget focused on programs for people with disabilities	–	–	3.3%
11. Percentage of the disability gap among the sufficiently physically active population	–	–	–
12. Percentage of government programs using sport as an instrument for peace and coexistence	4.3%	–	1.3%
Key data point	Chile	Costa Rica	Ecuador
1. There is a recent national survey on sports practice and physical activity, stratified by gender and age.	Partially	Partially	Partially
2. Governmental rank of the national authority for sports and physical activity	Ministry	Autonomous Organ	Ministry
3. Physical education is an independent mandatory subject in primary and secondary education at the national level.	Partially	Partially	Partially
4. There are legal frameworks to promote, enforce, and oversee gender equality and non-discrimination in sports and physical activity.	Yes	Yes	Partially

Empty cells (–) mean that no relevant value could be reported regarding the respective indicator (see 57).

With respect to the implementation of the indicators—specifically, the feasibility of calculating the data and indicator values—the following results were obtained. As summarized in Table 3, we can observe that four indicators (I3, I4, I8, and I9) were successfully calculated by the three countries utilizing the formulae proposed in the Ibero-American indicators model. However, three indicators (I2, I5, and I11) could not be calculated by any country. Additionally, for two indicators (I1 and I12), Chile and Ecuador were able to provide data, whereas Costa Rica was not.

In comparison, Chile was able to implement 8 out of 12 indicators (66.6% implementation), Costa Rica 4 out of 12 indicators (33.3% implementation), and Ecuador 7 out of 12 indicators (58.3% implementation).

## 4 Discussion

In this paper, we present and discuss the findings of the first pilot phase for the Ibero-American indicators model and discuss its possible value.

### 4.1 Overall findings

All three countries were able to implement information regarding the four key data points. None of the countries of the first pilot phase was able to provide data for all the indicators. Chile was able to implement eight, Costa Rica four, and Ecuador seven out of the twelve indicators. Thus, Chile obtained most of the values using the formulae of the Ibero-American model, completing two-thirds, while Ecuador followed closely behind with one less value. In contrast, Costa Rica faced the greatest difficulties in calculating values for the indicators, completing only a third of them.

It is important to note that the indicators I3, I4, I8, and I9 were informed by all the countries. This underscores the significance of these indicators in governmental statistical documentation and physical activity surveillance data, as well as their relative ease of collection at the national level. Additionally, the Ibero-American indicators model enables an objective comparison of countries based on these findings.

As further elaborated in the limitations section, this first pilot implementation encompassed several issues and compromises (e.g., regarding standardization). Hence, the presented findings have to be considered within this scope.

In the following, we will elaborate on the specifics in this order: (a) Key data points; (b) Indicators provided by all three countries (I3, I4, I8, and I9); (c) Indicators provided by two countries (I1 and I12); (d) Indicators provided by one (I6, I7, and I10) or no country (I2, I5, and I11).

### 4.2 Key data points

Regarding the first key data point (recent national survey on sports practice and physical activity), all three countries were able to partially comply with its requirements, considering some limitations. That is, for all countries, the surveys are not representative of key age groups (under 18, 18–64, and over 65 years old), and its methodology, microdata, and basic tabulations are not publicly accessible. Additionally, for Costa Rica and Chile, the reference survey is over five years old (43, 44, 54–56).

The governmental rank of the national authority for sports and physical activity (second key data point) differs across the three countries: Chile and Ecuador designate Ministries (54, 56, 58, 59), while Costa Rica assigns this responsibility to an Autonomous Organ (that is, Instituto Costarricense del Deporte y la Recreación [ICODER], 55, 60). This distinction may reflect differences in institutional authority and cross-sector coordination capacity, with Ministries generally enjoying broader policy influence, whereas autonomous bodies may operate with greater continuity but less integration. As such, institutional rank may shape a country's capacity to lead national strategies, influence resource allocation, and coordinate multisectoral implementation, particularly in the absence of strong formal coordination mechanisms.

Regarding the third key data point (physical education is an independent mandatory subject in primary and secondary education at the national level), all three countries were able to partially comply with its requirements, indicating some limitations. That is, in Chile's curriculum, the subject is called “Physical Education and Health”, but it is unclear whether physical education is separate from health education. Additionally, the number of hours per week for the subject is not clearly defined (54, 61). In Costa Rica, physical education is mandatory but not independent, and the curriculum does not clearly define the number of weekly hours (55, 60). In Ecuador, physical education is mandatory but not fully independent, as it shares hours with cultural and artistic education (56, 62).

As for the fourth key data point (legal frameworks to promote, enforce, and oversee gender equality and non-discrimination in sports and physical activity), Chile has a positive legal framework, with a general protocol for preventing and addressing sexual harassment, abuse, discrimination, and mistreatment in sports, approved by Supreme Decree in 2020, and evidence shows it has been applied (54, 63). Similarly, Costa Rica has legal frameworks to promote gender equality in sports, and there is evidence that they are committed to design effective policies and programs (55, 64–66). Ecuador has legal frameworks for gender equality in sports, but no evidence was provided on their application, resulting in a partial compliance of the data point value (49, 56).

### 4.3 Indicators provided by all three countries

#### 4.3.1 Indicator three (I3)

The mentioned necessity for more standardized data acquisition is evident in our results for I3 (percentage of the population sufficiently physically active stratified by gender and age). In this case, Chile shows a significantly lower percentage compared to Costa Rica and Ecuador, with its value being more than three times lower than those of the other two countries. This discrepancy may be partly attributed to the demographic characteristics of its population, as well as variations in the questionnaire used across countries. However, since the specific questionnaire for Costa Rica and Ecuador was not accessible (whereas Chile provided theirs), nor was the microdata from the data collection process publicly available, it was not possible to confirm this with certainty (57). Therefore, this highlights a key opportunity for developing indicators: while incorporating national definitions, each indicator must also include a concept and calculation method that are consistent across the entire region. The discrepancies described here have to be considered in the following tentative analysis of the available data.

Costa Rica and Chile provided data from 2018 (44, 67), while Ecuador's data was slightly more recent (45). Additionally, all sources provided data solely for adults (that is, individuals aged 18 and above, or 19 and above in the case of Costa Rica). Notably, within the model's results, Ecuador displayed a considerably high percentage for I3, showing that nearly four-fifths of its population is sufficiently physically active. In comparison, the values for Costa Rica indicate that slightly more than three-fifths of its population meets the requirements of the model's indicator. Lastly, the values for Chile indicate that less than one-fifth of its population meets the criteria set by the model's indicator.

Internationally, there are notable differences in physical activity levels, particularly as global estimates show that 27.5% of adults (18) and 81% of adolescents (9) do not meet the 2010 WHO recommendations for physical activity (8, 68, 69). The research team of GoPA! (38) also report that worldwide, approximately 30% of adults are physically inactive. In Latin America, 39% of adults and 85% of adolescents fail to meet recommended physical activity levels (16–18). As a comparison to our results, the GoPA! country cards provide a physical activity prevalence value of 65% for Chile for individuals of 15 years and above (Encuesta Nacional de Salud of 2017; 38). This value is relatively similar to the 73% reported by Ferrari et al. (17), yet both differ significantly from the 18.7% of this pilot study. For Ecuador, the physical activity prevalence value is 56% for individuals of 18 years and above (Encuesta Nacional de Salud y Nutrición of 2012; 38). Here, the value is also relatively similar to the 63% reported by Ferrari et al. (17), and slightly more in line with the 78% found in this study. Lastly, for Costa Rica, the physical activity prevalence value is 64% for individuals of 18 years and above (Encuesta de Factores de Riesgo Cardiovasculares of 2010–2018; 38). Ferrari et al. (17) reported a value of 52%, whereas this study also found 64% (for individuals aged 19 and above; and, notably, with a different source, that is, 44, 57).

#### 4.3.2 Indicator four (I4)

For I4 (percentage of deaths related to physical inactivity), all three countries were able to provide data, which were derived from the GoPA! country cards (38), with a value of 8.3% for Chile, 11.4% for Costa Rica, 11.0% for Ecuador. When compared to the global estimate of 9%, Chile's figure is slightly lower, while those of Costa Rica and Ecuador are somewhat higher. These differences, though not large, may reflect underlying disparities in population-level physical activity patterns, health system performance, and the burden of noncommunicable diseases. The similarity between Costa Rica and Ecuador suggests potentially shared structural or behavioral risk factors. Conversely, Chile's slightly lower rate may be indicative of comparatively more effective public health strategies, different demographic profiles, or better access to preventive services. However, interpretation should be approached with caution, particularly given potential variations in monitoring systems, data quality, and methodological assumptions across countries.

#### 4.3.3 Indicator eight (I8)

Regarding the percentage of women in managerial positions within the national sports entity (I8; aligned with SDG 5—gender equality), the data show that Chile and Ecuador are approaching gender parity, with women holding close to or just above 50% of these roles (54, 56). This suggests that both countries have made notable progress toward gender-inclusive leadership, at least within national-level sports administration. In the case of Chile, this positive finding sets it apart from the gender inequality revealed by I9. Thus, while institutional progress has been made, this contrast highlights that broader societal or behavioral barriers to women's participation in physical activity persist.

In contrast, Costa Rica's lower value for I8—with women occupying just over one-third of managerial roles—points to ongoing challenges in achieving gender equity in decision-making positions (55). Together with its relatively low gender disparities in physical activity levels (I9), this result highlights that progress in one domain of gender equality does not necessarily imply gains in others.

While I8 and I9 address different aspects of gender disparity (leadership representation and population-level participation, respectively), together, they reveal the multidimensional nature of gender inequality in sport and physical activity. A country may advance in one area while lagging in another. For example, Costa Rica's comparatively low percentage of women in managerial positions (I8) contrasts with its relatively smaller gender gap in physical activity levels (I9), highlighting that equity in governance structures does not automatically translate into equitable participation outcomes, and vice versa. This disconnect underscores the importance of addressing gender equality at multiple levels, from institutional leadership to grassroots participation, and tailoring policy responses accordingly.

Moreover, I8 stands out from other indicators for its relative ease of data access and update potential. While many other indicators rely on complex or infrequent population-level data (e.g., I3, I4, and I9), I8 can be monitored more regularly through institutional records. Furthermore, for Chile, the indicator had not been calculated in advance by the Ministry of Sport, requiring an analysis of the organizational structure to identify executive positions and gender distribution. To fulfill the indicator, the Ministry prepared a report, which will be periodically updated and published (47, 54). This example illustrates how this indicator can become a practical entry point for institutional accountability and gender audits in sport governance.

Lastly, the case of I8 also illustrates how the indicator model can catalyze improvements, even in the process of data production. In Chile, the need to calculate this indicator prompted a new internal process to assess and publish gender-disaggregated data. This is a clear example of how the indicator model, beyond measurement, can stimulate organizational change and encourage transparency.

#### 4.3.4 Indicator nine (I9)

Another indicator with noticeable differences is I9 (percentage of the gender gap among the sufficiently physically active population). Here, Costa Rica and Ecuador show very similar values, whereas Chile's is more than double their value. The source for these values in each respective country is the same as for I3. That is, Costa Rica and Chile were able to provide data from 2018 (44, 67), while Ecuador's data was slightly more recent (45). Additionally, all sources provided data solely for adults (that is, individuals aged 18 and above, or 19 and above in the case of Costa Rica).

Globally, there are notable inequalities in the physical activity levels of women and men, particularly as “data show that in most countries, girls and women are less active than boys and men” (8, p. 2). For all three countries in this study, the findings that men are more physically active than women are consistent with results from other studies regarding Latin America (17, 19, 20). Compared to the other two, Chile's value of 42.9% is noticeably larger, indicating that Chilean men are considerably more physically active than Chilean women. Ferrari et al. (17) investigated physical activity and sedentary behavior in eight countries with data from the Latin American Study of Nutrition and Health. In this study, physical activity and sedentary behavior data were collected using accelerometers to provide objectively measured information on aspects such as prevalence of physical inactivity, socioeconomic and education level, as well as gender disparities. The findings of Ferrari et al. (17) also show Chilean men to be more physically active, however, with a smaller difference than observed in our study. Contrarily, the authors reported noticeably higher differences of physical activity between men and women for both Costa Rica and Ecuador, whereas the gender difference values for both countries in this study were significantly lower (that is, less than half) than Chile's.

As a comparison, applying the calculation of gender disparity of our I9 (see Table 1) to the data provided by Ferrari et al. (17) on the prevalence of physical (in-)activity for men and women would result in a gender disparity value of ca. 10 percent for Chile, ca. 40 percent for Costa Rica, ca. 33 percent for Ecuador, and ca. 28 percent for the whole of the eight countries investigated in the Latin American Study of Nutrition and Health (see 17). Hence, there is a noticeable discrepancy between their accelerometer-based activity measures and our findings derived from national surveys, where data was self-reported (see Table 3). Notably, the relationship between higher and lower gender gap values is the opposite in these studies.

Furthermore, applying the calculation of gender disparity of I9 to the data provided by the GoPA! country cards results in a gender gap value of 25.9% for Chile for individuals of 15 years and above (Encuesta Nacional de Salud of 2017; 38). This value is significantly lower than the 42.9% of this pilot study, but higher than the 10% from Ferrari et al. (17). For Ecuador, the gender gap value is 41.3% for individuals of 18 years and above (Encuesta Nacional de Salud y Nutrición of 2012; 38). Here, the value is higher than the 33% reported by Ferrari et al. (17), and significantly higher than the 17% found in this study. Lastly, for Costa Rica, the gender gap value is 16.9% for individuals of 18 years and above (Encuesta de Factores de Riesgo Cardiovasculares of 2010–2018; 38). Interestingly, this value is very similar to the 15.3% of this study, and both are notably lower than the 40% from Ferrari et al. (17). All these commonalities and differences highlight the significant potential for future data collection and research regarding standardizing and refining criteria. Such efforts would enhance the comparability of data across studies and between nations.

With regard to I9 (linked to SDG 5, gender equality), it can also be observed in most countries in the region that the gender gap increases significantly with age (57, 70). One of the most important goals of sustainable development is to eliminate gender gaps or differences in their multiple dimensions. Therefore, it is crucial that governments and the public have indicators that allow them to monitor their progress toward reducing or eliminating the physical activity gap between men and women.

Overall, the notably higher gender gap in Chile, as well as discrepancies across data sources and countries, suggest that gender-responsive policies for physical activity promotion are either underdeveloped or unevenly implemented. For Chile, the large gap may indicate a need for more targeted interventions aimed specifically at increasing physical activity among women—particularly in adult and older age groups. Meanwhile, Costa Rica and Ecuador could examine, how existing policies or social norms have contributed to narrower gaps, and how to sustain or further reduce these differences. For all countries, the findings highlight the need to integrate disaggregated indicators like I9 into national monitoring frameworks, in line with SDG 5, and to design tailored interventions that address gender and age-specific barriers to physical activity.

### 4.4 Indicators provided by two countries

#### 4.4.1 Indicator One (I1)

For I1 (percentage of the national public budget invested in sports and physical activity), Chile and Ecuador were able to provide values, but Costa Rica was not. In 2023, 0.62% of Chile's public budget was allocated to the Ministry of Sport, the national governing body for sports and physical activity. A key challenge in calculating this indicator was the significant budget increase that year due to Chile hosting the Santiago 2023 Pan American and Parapan American Games. Typically, the Ministry's annual budget represents only 20% of the funds allocated in 2023 (54).

As for Ecuador, the allocated budget value was 0.23% for 2023 (42). During the process of generating the value for this indicator, the main challenge was reaching a consensus on what qualifies as investment in sports and physical activity, as the sectoral ministry does not necessarily account for all public spending in this area. Investments may also be allocated through decentralized autonomous governments. Additionally, within the current methodology, a key difficulty was identifying reliable data sources that offer a year-over-year view of public investment, ensuring alignment between the approved budget, planned allocations, and actual expenditures (56).

As mentioned above, Costa Rica was not able to provide a value for this indicator. A key challenge in its public policy is integrating sports and physical activity into the government's budget priorities, requiring ongoing advocacy to highlight their role in social development and public health. Strengthening ICODER's management is essential for securing more government funding. However, the involvement of multiple stakeholders has made data collection difficult, hindering effective analysis. Overcoming this challenge is crucial for improving sports policies and programs (55).

Overall, the allocation of the budget for sports and physical activity serves as a clear indicator of the sector's importance within public investment priorities and provides a concrete reference for understanding the scope of related policies based on available resources. However, a limitation of the current indicator is that it does not account for the funds allocated to sports and physical activity by government offices outside the national governing body. To improve accuracy, it would be beneficial to develop a broader indicator that captures the full amount of public resources allocated to the sector at different levels (57).

#### 4.4.2 Indicator twelve (I12)

Similar to I3, very salient differences can be observed among the countries for I12 (percentage of government programs using sport as an instrument for peace and coexistence). Here, Chile reports a value three times higher than that of Ecuador. To some extent, these differences may stem from variations in the countries' categorization and accounting systems. Thus, it is important to note that these indicator values may not be strictly comparable between countries, as Chile refers to “programs”, while Ecuador uses “projects” as its reference. Additionally, Chile classifies programs into different government categories than Ecuador, which can significantly impact the reported percentage levels (57). For instance, Chile's programs include “Protection of Specialized Programs for Children and Adolescents in Street Situations”, “Program for Children and Adolescents of Specialized Comprehensive Intervention”, and “Life Skills Program” (51); and Ecuador has a project called “Active Meeting of Sports for Development 2022–2025” (71).

### 4.5 Indicators provided by one (I6, I7, and I10) or no country (I2, I5, and I11)

Regarding the indicators with only one or no country providing a value, we will discuss the challenges encountered and, where applicable, their possible implications.

Only Chile provided data for I6 (percentage of school students sufficiently physically active stratified by gender). It is important to note that none of the existing national surveys referred by the technical teams considered the definitions of the WHO (8) regarding sufficient physical activity levels. However, through the efforts of the technical and consulting teams, in combination with information from the “Information System on Educational Trends in Latin America” (72), the data of the “National Survey on Physical Activity and Sports for Children Aged 5–17” (46) could be verified and applied to this indicator (54). The value 16.5% is notably lower than the prevalence of overall physical activity reported in Chile's 2022 Report Card on Physical Activity, where 37.0% of children and adolescents were considered physically active (73, 74).

Similar to I6, only Chile was able to provide a value for I7 (percentage of the national sport entity's budget focused on gender programs). The Chilean Ministry of Sport does not have government programs specifically focused on gender issues, which makes it impossible to allocate a distinct budget for them, resulting in a value of 0%. However, the ministry implements strategies to reduce the gender gap in sports, such as offering women-only physical activity workshops, funding women's soccer development, and supporting seasonal agricultural workers by providing physical activity workshops for their children (54). Similarly, while the Ministry of Sport of Ecuador undertakes various gender-related initiatives, it lacks a defined framework for allocating resources to sports organizations based on this focus. As a result, funding data is not disaggregated (56).

For I10 (percentage of the national sport entity's budget focused on programs for people with disabilities), only Ecuador was able to provide a value. This indicator was the result of internal work of the technical team to define what could be considered as investment amounts related to disability, establishing criteria for considering resources for investment projects and current spending (56). The Chilean Ministry of Sport does not seem to have exclusive programs for people with disabilities, making it difficult to define a specific budget for such initiatives. However, it implements various strategies within broader government programs to benefit people with disabilities, such as supporting athletes, funding the Chilean Paralympic Committee, creating competition spaces for Paralympic athletes, and financing related sports projects and research (75). Also, there is a fraction of the budget at the National Disability Service allocated to fund inclusive projects, with a specific area of inclusive sports. These efforts contribute to the development of this population within existing programs (54).

Lastly, regarding the indicators, no country was able to provide data for: To calculate a value for I2 (percentage of the contribution of sports and physical activity to the national GDP), a country must have a satellite account, which is not available for Chile, Costa Rica, nor Ecuador. For I5 (percentage of schools providing the minimum number of minutes of physical education), verified administrative records are necessary for its calculation, which neither of these countries was able to provide. Similarly, for I11 (percentage of the disability gap among the sufficiently physically active population), a survey is required for calculation, but neither of these countries reported surveys with data representative for people with disabilities (57).

Overall, for I2, I5, I6, I7, I10, and I11, the limited availability of data, with either no values or only a single value calculated, represents a significant blind spot in the analysis. As observed, the model's ability to enable meaningful cross-country comparisons is reduced in the context of such data gaps. However, these limitations also serve as diagnostic tools, highlighting areas of systemic weakness that may prompt corrective action. Specifically, they underscore the need to strengthen national information systems through increased investment in data acquisition and improved surveillance mechanisms. Additionally, the absence of data for the indicators signals the necessity of policy adjustments to address underlying structural gaps and to ensure more comprehensive and consistent reporting in the future.

Beyond diagnosing current limitations, the Ibero-American indicators model also offers strategic potential. As a standardized yet iteratively adaptive framework, it enables governments to assess both the alignment of their public policies with the SDGs (policy awareness) and their practical capacity to implement and measure them (policy effectiveness). In doing so, it exposes critical mismatches—such as indicators featured in policy documents but lacking measurable data, or those with available data but absent from official policy agendas—and provides a roadmap for evidence-informed policy reform and improved SDG integration (see 76).

## 5 Limitations and future efforts

The first national pilot implementation of this project identified several challenges in the operational processes for generating indicators. As such, we would like to emphasize that all these comparisons are based on pilot data from these countries, which are highly likely to be subject to increased variance or dispersion due to factors such as the date of data collection, the validity of the source (e.g., regarding standardization), the data sample and population, and differences in measurement and data processing. Therefore, the comparisons presented in this paper, where we elaborate on specific values, must be considered within this scope of accuracy and specificity. Accordingly, the findings should be interpreted with caution, considering the following limitations:

Key issues included significant variability in administrative and accounting categories across countries, which hindered the comparability of indicators. There was also limited documentation of processes, insufficient reliable data sources, and lack of public access to survey methodology and microdata, impeding transparency and external validation. Another limitation was the lack of recent surveillance data to inform physical activity indicators. The model requires surveys to be no older than five years, yet some used in this study dated back to 2018, limiting their ability to capture recent policy impacts. The fragmentation of information presented another obstacle. Additionally, future implementations of these indicators should involve a broader team of experts in research and practice, who can enhance the identification of relevant data to inform the indicators. However, the strong political commitment demonstrated by the countries offers an opportunity to improve these operational processes and enhance the generation of more reliable and comparable indicators in the future (57).

Regarding future research and public policy efforts, increased public investment is necessary to enhance the technical capacities of administrative teams involved in data acquisition, indicator calculation, process documentation, and raising awareness of currently unmeasurable indicators. If countries progressively expand, adapt, and standardize their data collection methods, future data will become much more comparable, making analyses using the Ibero-American indicators model increasingly valuable. Furthermore, maintaining a standardized dataset, as proposed by the model, can track the efforts and progress made by governments. Standardization across countries in the region would foster productive debates, enabling the comparison and sharing of strategies to advance the promotion of the SDGs.

In their article on evidence-based physical activity interventions and their real-world application in policy and practice, Ramirez Varela et al. (79) argue that simply disseminating results through scientific papers and other media is insufficient to influence real-world physical activity policy and practice. They emphasize the importance of leading efforts in co-creation, co-dissemination, advocacy, and capacity building to better engage policymakers and communities. Additionally, they recommend that funders of physical activity research support projects that integrate high-quality research with dissemination both within and beyond academic circles (79). In line with these recommendations and as part of their ongoing efforts, the consortium of the Ibero-American indicators model hosted an international training course in Madrid (in June 2025) to share the latest progress with researchers, stakeholders, and other interested parties.

As part of the GoPA! team, Ramirez Varela and Hallal (77) emphasize the need to differentiate physical activity based on purpose and context, not just its domain (e.g., leisure and work). They argue that distinguishing between activity done out of necessity vs. by choice is crucial for accurate assessment (see 78). According to the authors, relying solely on physical inactivity indicators is inadequate; a more comprehensive approach is needed, including additional indicators for global physical activity data. They also stress tailoring recommendations for individuals in high-risk occupations to achieve health-enhancing physical activity goals (77). In this context, the current set of Ibero-American indicators does not differentiate between types of physical activity, such as leisure or work-related activity. Instead, it aligns with the WHO's guidelines, which state that “all physical activity counts [and that] physical activity can be done as part of work, sport and leisure or transport (walking, wheeling and cycling), as well as every day and household tasks” (8, p.1). Nevertheless, the remarks by Ramirez Varela and Hallal (77) are valuable and valid and should be considered in future assessments.

## 6 Conclusion

### 6.1 Key findings

The first national pilot implementation of the Ibero-American indicators model highlights both its potential and its limitations as a tool for monitoring and evaluating physical activity and sport policies in relation to the SDGs.

Although none of the three participating countries were able to report data for all twelve indicators, partial coverage was achieved: Chile reported on eight indicators, Ecuador on seven, and Costa Rica on four. Notably, all three countries provided data for four indicators (I3, I4, I8, and I9), suggesting these are well embedded in national statistical systems and relatively feasible to collect. These results underscore the model's potential for enabling cross-country comparisons and tracking policy outcomes over time.

Nevertheless, key challenges persist. These include inconsistent administrative procedures, outdated or unavailable surveillance data, limited access to methodological documentation, and a general lack of public microdata. Such issues hinder the model's comparability and broader policy relevance.

### 6.2 Recommendations

To unlock the full potential of the Ibero-American indicators model, countries should work toward strengthening institutional coordination to ensure more coherent data practices across sectors. This should be accompanied by greater investment in technical capacities to support reliable, timely, and disaggregated data collection. Furthermore, efforts should be made to standardize methodologies and promote transparency by publishing data sources and protocols. Finally, expanding access to recent and high-quality surveillance data—particularly for underdeveloped indicators—will be essential to improve the model's policy relevance and comparability.

By addressing these areas, the indicator model can more effectively guide evidence-based policymaking and foster meaningful regional dialogue around physical activity and sport as drivers of sustainable development.

## Data Availability

The datasets presented in this study can be found in online repositories. The names of the repository/repositories and accession number(s) can be found below: Zenodo: https://doi.org/10.5281/zenodo.15126424.
